# Infusion of Sibling Marrow in a Patient with Purine Nucleoside Phosphorylase Deficiency Leads to Split Mixed Donor Chimerism and Normal Immunity

**DOI:** 10.3389/fped.2017.00143

**Published:** 2017-06-19

**Authors:** Laura Yeates, Mary A. Slatter, Andrew R. Gennery

**Affiliations:** ^1^Institute of Cellular Medicine, Newcastle University, Newcastle Upon Tyne, United Kingdom

**Keywords:** purine nucleoside phosphorylase, hematopoietic stem cell transplantation, sibling donor, unconditioned, immune reconstitution

## Abstract

Purine nucleoside phosphorylase (PNP) deficiency, a rare autosomal recessive metabolic disease causes combined immunodeficiency and developmental delay, hypotonia, and spasticity. Patients present with recurrent infections associated with T-lymphocytopenia, characteristically presenting later than patients with classical severe combined immunodeficiency (SCID). PNP, with adenosine deaminase (ADA), is part of the purine salvage pathway. The only curative therapy is hematopoietic stem cell transplantation. Myeloablative conditioning is recommended to prevent rejection caused by residual immune function. However, HLA-identical sibling stem cell infusions in ADA-SCID result in some donor stem cell engraftment and long-term thymopoiesis. We report a patient with PNP deficiency, who received HLA-identical sibling marrow without chemotherapy because of disseminated cytomegalovirus (CMV) infection. The patient presented at 14 months of age following recurrent infections, from early infancy, with persistent irritability, developmental delay, and hypotonia. She had neutropenia, pan-lymphocytopenia, and hypogammaglobulinemia with low plasma urate and erythrocyte PNP activity. Diagnosis was confirmed with a homozygous mutation in *PNP*. The patient was viremic with CMV detected in blood and CSF by PCR. Dual antiviral therapy improved the clinical condition and reduced the viral load. In view of the disseminated CMV infection, the decision was made to infuse stem cells without any pre-conditioning chemotherapy. She received a matched sibling donor unconditioned stem cell infusion at 16 months of age. The post-transplant course was uneventful. Blood PCR became negative for CMV. Global hypotonia persisted, although with significant improvement in irritability. At 4 years of age and 29 months post-transplant, the patient demonstrated normal T-lymphocyte and natural killer cell numbers. Recent thymic emigrants represented 12% of the total T-lymphocyte population. Lymphocyte proliferative responses to phytohemagglutinin were normal. Memory and class-switched B-lymphocytes were present. Immunoglobulin replacement had been discontinued, and there were normal IgG responses to tetanus vaccine, *Haemophilus influenzae* type B and pneumococcal conjugate vaccine antigens. There was 93% donor T-lymphocytes, 20% donor B-lymphocytes, and 5% donor myeloid cells, indicative of some donor stem cell engraftment. There was no significant infection history despite regular nursery attendance. Height and weight were following the 50th centile. Split mixed donor chimerism has corrected the immunological defect.

## Introduction

Purine nucleoside phosphorylase (PNP) deficiency (OMIM 613179) is a rare autosomal recessive metabolic disease leading to combined immunodeficiency and neurological abnormalities, which may include developmental delay, hypotonia, and spasticity (1, 2). Autoimmune manifestations are also reported, predominantly hematological cytopenias, but arthritis and systemic lupus erythematosus are reported (3). Patients present with predominantly T-lymphocytopenia, and the immunological presentation characteristically is later than patients with classical severe combined immunodeficiency (SCID) (4). Infectious complications include recurrent otitis media and lower respiratory tract infections—*Pneumocystis jirovecii* pneumonitis is a recognized presentation. Mucosal candidias is well documented, and patients can also present with disseminated BCG infection if they have been vaccinated. Susceptibility to viral infections including herpes viruses and polyoma JC virus is recognized (4–6).

Purine nucleoside phosphorylase is a ubiquitously expressed enzyme of the purine salvage pathway as is adenosine deaminase, deficiency of which also causes immunodeficiency. PNP is expressed at high levels in lymphocytes and causes the release of ribose phosphate by converting inosine to hypoxanthine, guanosine to guanine, and deoxyguanosine to guanine. PNP deficiency results in the accumulation of deoxyguanosine, which is converted to deoxyguanosine triphosphate (dGTP) by deoxycytidine kinase specific to lymphocytes. The excess of dGTP inhibits ribonucleotide reductase and DNA synthesis, resulting in lymphocytotoxicity, affecting primarily T-lymphocytes possibly due to the proliferative stress to which thymocytes are subject during T-lymphocyte differentiation (7–9).

Neurological and infectious manifestations predominate in the clinical presentation, and patients usually present after the first year of life, in contra-distinction to adenosine deaminase-deficient patients. All patients have elevated inosine, deoxyinosine, guanosine, and deoxyguanosine in blood and urine. The blood urate is typically low. Patients demonstrate B- and T-lymphocytopenia (CD4 > CD8) with poor proliferative responses, but normal numbers of NK lymphocytes. Panhypogammaglobulinemia is usually present, with absent vaccine-specific antibodies.

Supportive treatment in the form of immunoglobulin replacement and antimicrobial prophylaxis should be initiated at diagnosis. However, the only curative therapy is hematopoietic stem cell transplantation (1). Some form of myeloablative conditioning regimen is generally recommended to counter the risk of rejection caused by residual immune function, which has been documented (10–13).

Long-term immune reconstitution in patients with SCID generally requires thymopoiesis to generate a normal, long-lasting naïve T-lymphocyte repertoire, best achieved if donor myeloid chimerism is present (14, 15). This, in turn, generally requires some chemotherapy to open the marrow niche to donor hematopoietic stem cells. Uniquely, patients with adenosine deaminase-deficient SCID can achieve partial donor stem cell engraftment following infusion of donor inoculum harvested from an HLA-identical sibling (16). It is thought that “auto-conditioning” creates some marrow stem niches through the effects of toxic purine metabolites, thus enabling some donor hematopoietic stem cell engraftment. We report a patient with PNP deficiency, in whom we infused HLA-identical sibling marrow without chemotherapy because of disseminated cytomegalovirus (CMV) infection, and in whom split mixed donor chimerism has corrected the immunological defect.

## Patient and Methods

The patient, the second child of consanguineous Kurdish parents, born at term with an uneventful antenatal and postnatal course, presented at 14 months of age. She did not receive BCG vaccination but was otherwise fully vaccinated. There was a history of recurrent infections, from early infancy. She was persistently irritable, feverish, with developmental delay and hypotonia, with symptoms developing from 1 month of age. There was no family history of immunodeficiency or neurological problems.

Immunological investigations revealed neutropenia (0.18 × 10^9^/l) pan-lymphocytopenia (Table 1) and hypogammaglobulinemia (IgG < 1.09 g/l, IgM < 0.05 g/l). The plasma urate was 69 µmol/l (100–260). Erythrocyte PNP enzyme activity was low at 1,967 μmol/l (normal 3,000–7,000 μmol/l) and a homozygous mutation in *PNP*, exon 2, c.59A>C, p.20H>P was identified confirming PNP-deficient immunodeficiency.

**Table 1:** Immunological parameters before and after hematopoietic stem cell transplantation.

	Pre-transplant	Post-transplant				1.92 years	3.42 years
		D + 18	D + 53	D + 102	D + 203		
**Lymphocyte subsets**							
CD3	12	994	438	1,877	6,621	3,239	2,065
CD8	9	703	204	1,331	5,551	2,164	1,053
CD4	3	296	218	518	1,013	996	925
NK	52	40	52	122	283	147	139
CD19	9	26	9	72	146	106	92
DR+ (%)	–	84	70	86	77	54	46
CD45RA/CD27^+^	–	20	–	–	728	586	372
CD19^+^/CD27^+^/IgM^−^	–	–	–	–	–	6%	9%
**Chimerism (%)**							
Donor CD3	–	96	96	100	100	93	93
Donor CD19	–	–	–	–	–	–	20
Donor CD15	–	5%	10	6	5	5	5
**Immunoglobulins**							
IgG	<0.3	9.4^a^	16.6^a^	7.2^a^	9.0^a^	3.4	4.7
IgA	<0.04	<0.04	<0.04	0.15	0.43	0.37	0.55
IgM	<0.05	<0.50	0.05	0.25	0.95	0.24	0.41
Tetanus	0.04	–	–	–	–	0.18^b^	4.26^c^
Hib	<0.01	–	–	–	–	0.5^b^	>9.0^c^

*^a^On IVIG*.

*^b^Pre-vaccination, off IVIG replacement*.

*^c^Post-vaccination*.

The patient was discovered to be viremic with CMV 10^7^ copies/ml in blood by PCR and CMV in the cerebrospinal fluid.

She received dual therapy for disseminated CMV (with CMV encephalopathy) with gancilovir 10 mg/kg, increased from the initial dose of 5 mg/kg due to the high viral loads and CMV encephalopathy, and with foscarnet. *Aspergillus fumigatus* was isolated from broncho-alveolar secretions and treated with liposomal amphotericin and voriconazole. She commenced regular immunoglobulin therapy and granulocyte colony stimulating factor to treat her neutropenia. Immunologically T-B-NK + SCID was confirmed.

With dual anti-viral therapy, she clinically improved, although remained very hypotonic and was irritable at times. Her CMV viral load in blood slowly reduced to 10^3^ copies/ml at the time of transplant.

## Results

An older, asymptomatic CMV positive HLA-identical sibling was chosen as a donor. In view of the disseminated CMV infection, the decision was made to infuse stem cells without any pre-conditioning chemotherapy. The parents were counseled that this procedure would clear the CMV infection, but a further conditioned transplant would likely be required. She received a red cell depleted matched sibling donor unconditioned stem cell infusion at 16 months of age, with the aim of gaining peripheral T-lymphocyte engraftment and control of the CMV infection.

The inoculum contained 62 ml of bone marrow with 3.6 × 10^8^/kg nucleated cells, CD34 6.7 × 10^6^/kg, CD3 5.0 × 10^7^/kg, and CD19 2.2 × 10^7^/kg without conditioning. She received ciclosporin as graft-versus-host disease (GvHD) prophylaxis from day −3.

The post-transplant course was largely uneventful. Grade 1 skin GvHD responded to topical steroid treatment. Blood PCR became negative for CMV. Global hypotonia persisted, although there was a significant improvement in irritability. It was unclear how much the CMV infection contributed to the hypotonia, which is classically associated with PNP deficiency.

At 4 years of age and 29 months post-transplant the patient demonstrated full immune reconstitution. T-lymphocyte and natural killer cell numbers were normal. Recent thymic emigrants, denoted by CD4^+/−^CD45RA^+^CD27^+^ were present as 18% of the total T-lymphocyte population. Lymphocyte proliferative responses to phytohemagglutinin were normal. B-lymphocytes were slightly below the normal range, but memory and class-switched B-lymphocytes present (Table 1). Immunoglobulin replacement had been discontinued. IgM was slightly below the normal range, but IgA and IgG were normal and there were normal IgG response to tetanus vaccine antigens, as well as to *Haemophilus influenzae* type B and the 13-valent pneumococcal conjugate vaccine antigens. There was no evidence of CMV in blood by PCR. Autoantibodies were absent. Chimerism analysis demonstrated 93% donor T-lymphocytes, 20% donor B-lymphocytes and 5% donor myeloid cells, indicative of some donor stem cell engraftment. The plasma urate and erythrocyte PNP enzyme activity remained low at 64 and 1,967 µmol/l, respectively. There was no significant infection history despite regular nursery attendance. Height and weight were following the 50th centile.

Neurological assessment confirmed mild to moderate developmental delay, with global hypotonia. The patient was able to articulate few words due to pharyngeal hypotonia, but had normal comprehension. She was able to sit unaided, stand, and walk with support.

## Discussion

Purine nucleoside phosphorylase deficiency is an autosomal recessive metabolic disease, which typically causes immune dysfunction, spasticity, and developmental delay. The only curative treatment to date is hematopoietic stem cell transplantation. This typically follows a period of conditioning with chemotherapy, before infusion from a matched donor. A previous report of an unconditioned infusion from an HLA-matched non-sibling family member in a patient with PNP deficiency reported donor T-lymphocyte engraftment with no B-lymphocyte (and presumably no stem cell) engraftment (17). Failure of stem cell engraftment may have been due to the inflammatory mileau caused by pre-existing BCG infection. Our case demonstrated some hematopoietic stem cell engraftment, which enabled enough T- and B-lymphocyte development to cure the underlying immunodeficiency, given the selective advantage of lymphocytes with normal enzyme activity. As most stem cells remained of recipient origin, the plasma urate and erythrocyte enzyme activity was unaltered. The improvement in neurological status was most likely due to clearance of CMV, as there is no good evidence that hematopoietic stem cell transplantation can reverse the neurological sequalae in PNP deficiency, even with full donor chimerism.

Adenosine deaminase-deficient SCID patients have an improved outcome when undergoing unconditioned transplant compared to those whom have received myeloblative therapy (16). Unusually for patients receiving a stem cell infusion, these patients demonstrate some donor myeloid chimerism. The likely explanation for this is that there is a degree of “autologous conditioning” by the accumulated deoxyadenosinetrisphosphate (dATP), which causes inhibition of ribonucleotide reductase, necessary for DNA replication and repair, as well as inducing caspase-mediated apoptosis through interaction with cytoplasmic apoptotic protease-activating factor 1 (Apaf-1) and cytochrome *c*. These cytotoxic effects may create some space in the stem cell niche to facilitate a degree of donor stem cell engraftment.

Purine nucleoside phosphorylase not only participates in the same purine salvage pathway as adenosine deaminase but also leads to an accumulation of dGTP rather than dATP. Like dATP, dGTP inhibits ribonucleotide reductase and DNA synthesis and repair. Additionally, there is experimental animal model evidence that the mitochondrial accumulation of dGTP initiates apoptosis by interference with the repair of mitochondrial DNA damage (18).

Thus, there is a hypothetical reason to suspect that patients with PNP deficiency may undergo a degree of autologous conditioning, analogous to those patients with adenosine deaminase deficiency. While we based our decision to omit chemotherapy on clinical grounds, and had counseled the parents that a fully conditioned transplant following clearance of CMV may be required, nevertheless, our rationale was partly based on reason given that PNP and adenosine deaminase are part of the same purine salvage pathway.

The outcome of patients with adenosine deaminase deficiency who receive an infusion is superior to those who receive conditioning (16). PNP deficiency is less common than adenosine deaminase deficiency. Nevertheless, a multicenter study may be able to determine outcomes of patients who did or did not receive conditioning. Furthermore, it would be important to try and determine whether the degree of myeloid chimerism was important in determining neurological as well as immunological outcome. Of course, chemotherapy may have an adverse effect in terms of neurological development, particularly on a susceptible neurological substrate. Our case demonstrates at least that a sibling infusion can lead to tri-lineage mixed donor chimerism and normal immunological function.

## Ethics Statement

This study was carried out in accordance with the recommendations of “The Royal Victoria Infirmary IRB” with written informed consent from all subjects. All subjects gave written informed consent in accordance with the Declaration of Helsinki.

## Author Contributions

AG and MS conceived the manuscript; LY gathered the data and wrote the manuscript; and all authors contributed to the final version.

## Conflict of Interest Statement

The authors declare that the research was conducted in the absence of any commercial or financial relationships that could be construed as a potential conflict of interest.
